# Effects of exposure length, cortical and trabecular bone contact areas on primary stability of infrazygomatic crest mini-screws at different insertion angles

**DOI:** 10.1186/s12903-024-04626-7

**Published:** 2024-08-09

**Authors:** Bingran Du, Yuan Lin, Mohan Ji, Qiaohua Yang, Jiang Jiang, Fei Wang, Xiaoyi Wang, Jinchuan Tan, Rui Jia, Jianyi Li

**Affiliations:** 1 Department of Stomatology, Shunde Hospital, Southern Medical University (The First People’s Hospital of Shunde, Foshan), Foshan, Guangdong 528308 China; 2 Stomatological Hospital, School of Stomatology, Southern Medical University, Guangzhou, Guangdong 510515 China; 3 Department of Ultrasound, The Shenzhen Second People’s Hospital, Shenzhen, 518035 China; 4 Department of Anatomy, Guangdong Provincial Key Laboratory of Digital Medicine and Biomechanics, Guangdong Engineering Research Center for Translation of Medical 3D Printing Application, National Virtual and Reality Experimental Education Center for Medical Morphology, School of Basic Medical Sciences, Southern Medical University, Guangzhou, 510515 China; 5 The Department of Anatomy, Nanchang Medical College, Nanchang, 330052 China; 6 The Department of Stomatology, The Seventh Affiliated Hospital, Southern Medical University, Foshan, Guangdong 528244 China; 7 Department of Rehabilitation Medicine, Guangdong Provincial People’s Hospital, Guangdong Academy of Medical Sciences, Southern Medical University, No. 106 Zhongshan Road II, Guangzhou, 510080 P. R. China

**Keywords:** Bone, Stability, Mini-screws, Insertion angles

## Abstract

**Background:**

The infrazygomatic crest mini-screw has been widely used, but the biomechanical performance of mini-screws at different insertion angles is still uncertain. The aim of this study was to analyse the primary stability of infrazygomatic crest mini-screws at different angles and to explore the effects of the exposure length (EL), screw-cortical bone contact area (SCA), and screw-trabecular bone contact area (STA) on this primary stability.

**Methods:**

Ninety synthetic bones were assigned to nine groups to insert mini-screws at the cross-combined angles in the occlusogingival and mesiodistal directions. SCA, STA, EL, and lateral pull-out strength (LPS) were measured, and their relationships were analysed. Twelve mini-screws were then inserted at the optimal and poor angulations into the maxillae from six fresh cadaver heads, and the same biomechanical metrics were measured for validation.

**Results:**

In the synthetic-bone test, the LPS, SCA, STA, and EL had significant correlations with the angle in the occlusogingival direction (*r*_*LPS*_ = 0.886, *r*_*SCA*_ = -0.946, *r*_*STA*_ = 0.911, and *r*_*EL*_= -0.731; all *P* < 0.001). In the cadaver-validation test, significant differences were noted in the LPS (*P* = 0.011), SCA (*P* = 0.020), STA (*P* = 0.004), and EL (*P* = 0.001) between the poor and optimal angulations in the occlusogingival direction. The STA had positive correlations with LPS (*r*_*s*_ = 0.245 [synthetic-bone test] and *r* = 0.720 [cadaver-validation test]; both *P* < 0.05).

**Conclusions:**

The primary stability of the infrazygomatic crest mini-screw was correlated with occlusogingival angulations. The STA significantly affected the primary stability of the infrazygomatic crest mini-screw, but the SCA and EL did not.

## Background

The infrazygomatic crest mini-screw has been widely used in orthodontic treatment, given its advantage in unobstructed tooth movement [1–3]. In clinical practice, an infrazygomatic crest mini-screw is recommended at a specific angulation close to the mucogingival junction to decrease the risk of soft tissue inflammation and root contact [1]. However, mobility of the infrazygomatic crest mini-screw can occur at the initial stage after insertion [4, 5], leading to root damage [6] and unnecessary burden associated with re-inserting the mini-screws [7]. Therefore, reducing the early mobility of infrazygomatic crest mini-screws is crucial for improving clinical treatment efficacy and post-treatment satisfaction.

Mobility of mini-screw at the initial stage is mainly related to insufficient primary stability [8]. Primary stability is the initial holding power of the mini-screw in the bone [9], which can be affected by the insertion angle [10–13]. Wu et al. reported the resistance strength of infrazygomatic mini-screws inserted at 90° into an artificial bone [14]. However, the insertion angle of 90° is quite different from that in real clinical settings, in which the infrazygomatic crest mini-screw should be inserted at a gingival tipping angle in the occlusogingival direction and distal tipping angle in the mesiodistal direction. Hence, it is necessary to provide more information on the primary stability of infrazygomatic crest mini-screws at different commonly used angles in the mesiodistal and occlusogingival directions.

The cortical bone thickness in contact with the mini-screw increases when the mini-screw is inclined towards the bone surface, thus improving the mechanical retention and stability [12]. The change in the screw-cortical bone contact area (SCA) may result in the primary stability differences of mini-screws at different angles. However, the cortical bone is not the only factor affecting the primary stability of mini-screws. Trabecular thickness has been reported to be correlated with the primary stability of mini-screws in fresh bovine pelvic bones [15]; therefore, the effect of the screw-trabecular bone contact area (STA) should not be ignored. Moreover, the mini-screw’s exposure length (EL) is another factor affecting primary stability because the Class II lever arm effect existed in the shear test [16]. Although many studies have realized the importance of these three abovementioned factors on the primary stability of mini-screws [15, 17–19], the specific effect of each factor on the primary stability of infrazygomatic crest mini-screws at different insertion angles has not been explored.

This study aimed to analyse the primary stability of the infrazygomatic crest mini-screw at different insertion angles in the occlusogingival and mesiodistal directions and to explore the effects of STA, SCA, and EL on the primary stability of infrazygomatic crest mini-screws. The null hypothesis was that no differences in the primary stability would exist among the mini-screws at different insertion angles and STA, SCA, and EL would have no effects on the primary stability.

## Materials and methods

### Synthetic-bone test of mini-screws at different angles

Synthetic bone blocks made of solid rigid polyurethane foam (Sawbones, Pacific Research Laboratories Inc., Vashon Island, WA, USA) were used for the experiments. A 1.5-mm rigid polyurethane foam sheet (simulating cortical bone, 40 PCF) attached to a 40-mm block (simulating cancellous bone, 15 PCF) was used as a bone model for the infrazygomatic crest region based on the reported cortical bone thickness [20]. The dimensions of each synthetic bone block were 20 × 20 × 41.5 mm^3^.

**Fig. 1:**
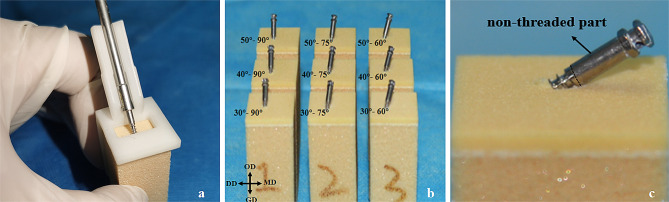
The insertion of the infrazygomatic crest mini-screw. (**a**) Customised guide plate for mini-screw insertion. (**b**) Nine groups with different combinations of the occlusogingival and mesiodistal angles. (**c**) The mini-screw was inserted until the beginning of the non-threaded part contacted the bone surface. *MD* mesial direction; *DD* distal direction; *GD* gingival direction; *OD* occlusal direction

Mini-screws (Ningbo Cibei Medical Treatment Appliance Co., Ltd, Zhejiang, China) of 2.0 mm in diameter and 13 mm in total length were inserted into the bone surface by the same operator with the help of a self-designed guide plate (Fig. 1a). The insertion protocol was consistent with the insertion method described in a previous study [21], but the final rotated insertion angle was decided based on the guide plate. Nine groups (3 × 3) were formed according to the cross-combinations of bone contact angles in the occlusogingival (30°, 40°, and 50°) and mesiodistal (90°, 75°, and 60°) directions (Fig. 1b). The bone contact angles in the occlusogingival and mesiodistal directions were designed according to previously reported studies [21–23], which included the commonly used angles in clinical practice. Each group comprised ten synthetic bone blocks, and the overall number of blocks was ninety (*n* = 90). The mini-screw was inserted into the blocks at different angles until the lower ends of the non-threaded parts touched the bone surface (Fig. 1c).

**Fig. 2:**
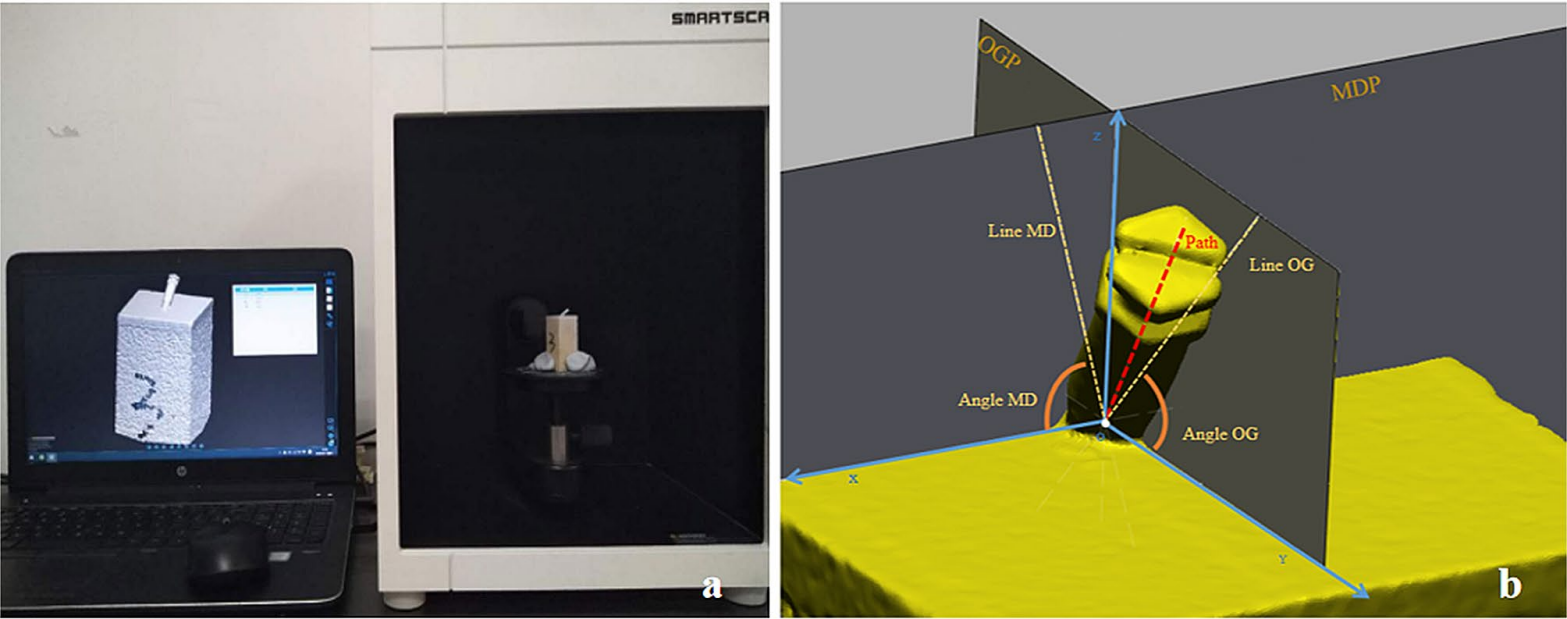
Measurement of the actual insertion angle of the mini-screw. (**a**) The 3D models obtained using SmartScan. (**b**) Measurement of the actual insertion angle of the mini-screw. *Angle MD* actual insertion angle of the mini-screw in the mesiodistal direction; *Angle OG* actual insertion angle of the mini-screw in the occlusogingival direction; *Line MD* the projection of the insertion path on the mesiodistal plane (MDP); *Line OG* the projection of the insertion path on the occlusogingival plane (OGP)

**Fig. 3:**
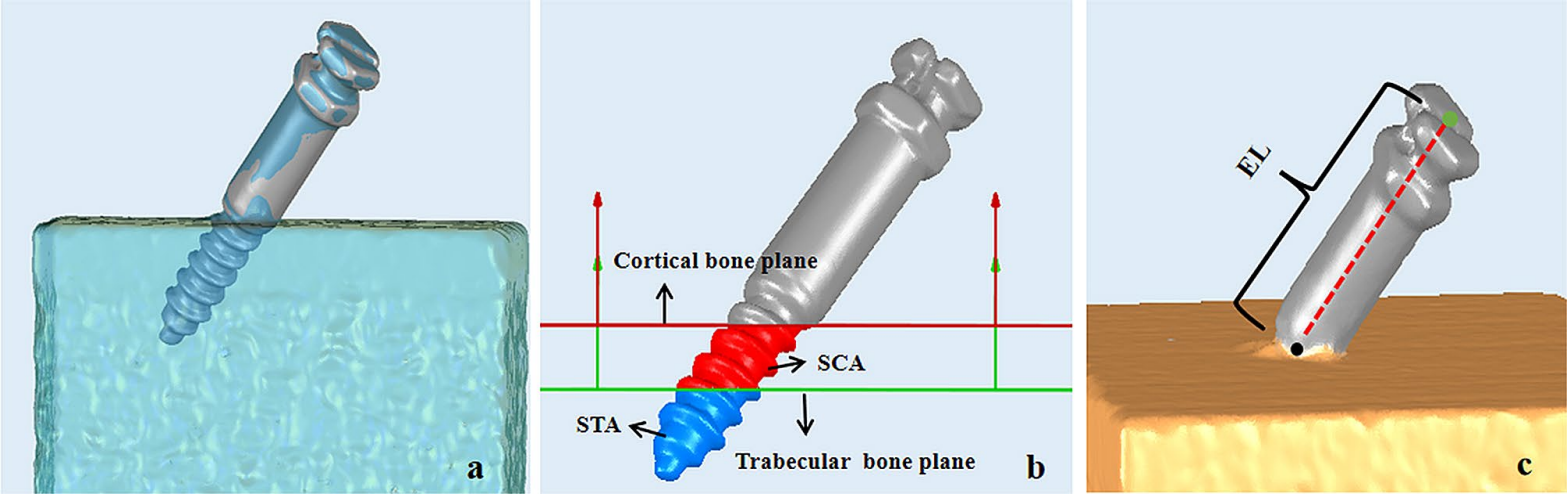
Measurement of the SCA, STA, and EL of the mini-screw. (**a**) The rendering of the two models after registration. blue model, the 3D model of screw-bone block; grey model, the 3D model of the mini-screw. (**b**) Measurement of the SCA and STA; blue area, STA; red area, SCA. (**c**) Measurement of EL; black point, the insertion site; green point, the apex of the mini-screw cap. *SCA* screw-cortical bone contact area; *STA* screw-trabecular bone contact area; *EL* exposure length

The 3D models of each screw-bone block and mini-screw were obtained using SmartScan (Guangzhou Electronic Technology Co., Ltd., Guangzhou, Guangdong, China) (Fig. 2a). Based on the 3D screw-bone model, Geomagic Studio 12.0 (3D Systems Inc., Rock Hill, SC, USA) was employed to simulate the insertion path of each mini-screw and create the mesiodistal and occlusogingival planes. These features were saved in Initial Graphics Exchange Specification format and imported into Geomagic Design X (3D Systems, Morrisville, NC, USA) to measure the actual insertion angle of the mini-screw (Fig. 2b). Then, the 3D mini-screw model and screw-bone model were fitted in Geomagic Studio 2014 (Fig. 3a). Subsequently, according to the cortical and trabecular bone the mini-screw passed through, cortical and trabecular bone planes were created using their software functions. SCA and STA were also calculated based on these planes (Fig. 3b). Finally, the EL−the distance of the insertion site to the apex of the mini-screw cap−was calculated by the “Compute Distance” function (Fig. 3c).

**Fig. 4:**
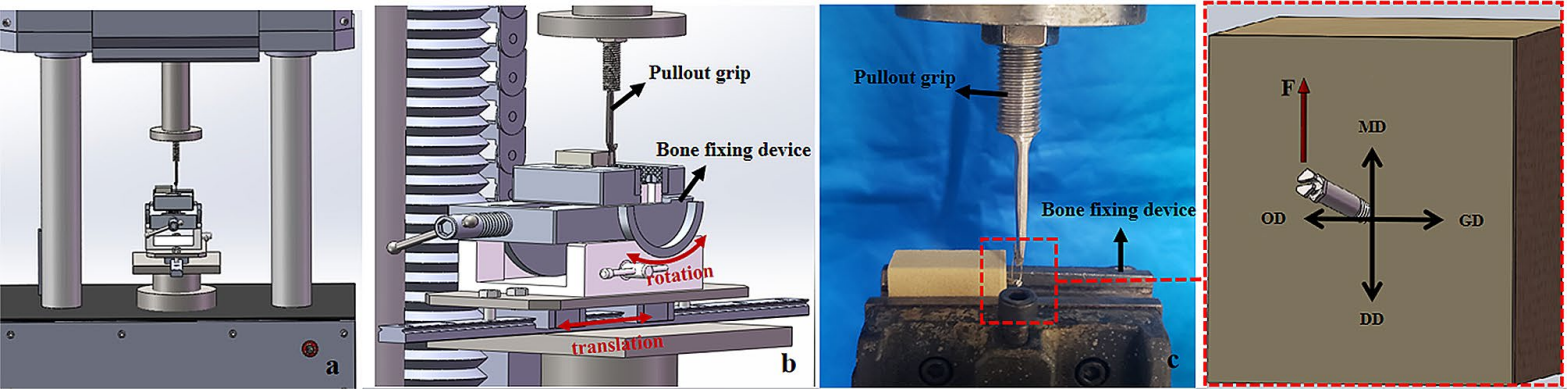
Lateral pull-out testing for the infrazygomatic crest mini-screw. (**a**) The mechanical testing machine for testing. (**b**) The pull-out grip and bone fixing device. (**c**) The procedure of lateral pull-out testing. *MD* mesial direction; *DD* distal direction; *GD* gingival direction; *OD* occlusal direction

Lateral pull-out testing was performed on the inserted mini-screws using a testing machine (ElectroForce 3510-AT, Bose Corp., Framingham, USA) at a constant speed of 0.05 mm/sec (Fig. 4a). In lateral pull-out testing, the customised pull-out grip and bone fixing device were specifically designed. The upper part of the lateral pull-out grip is directly fixed to the machine using stainless steel screws. The lower part is a J-shaped retaining arm (Fig. 4b). The bone fixing device can achieve forward and backward rotation and translation (Fig. 4b). Before lateral pull-out testing, a 0.5-mm orthodontic wire (Shanghai Dental Instrument Factory Co., Ltd., Shanghai, China) was passed through the hole of the mini-screw and tied to the J-shaped retaining arm. The traction was parallel to the bone surface and oriented in the mesial direction (Fig. 4c). Pull-out strength–displacement data were obtained, and the peak strength of each mini-screw was recorded in Newtons.

### Cadaver-validation test of mini-screws at the optimal and poor angulations

A verification experiment on fresh cadaver specimens was performed to further verify the effects of SCA, STA, and EL on the primary stability of mini-screws at different angles. The Medical Ethics Committee of our institution approved this verification experiment (Approval No. 2023-04).

**Fig. 5:**
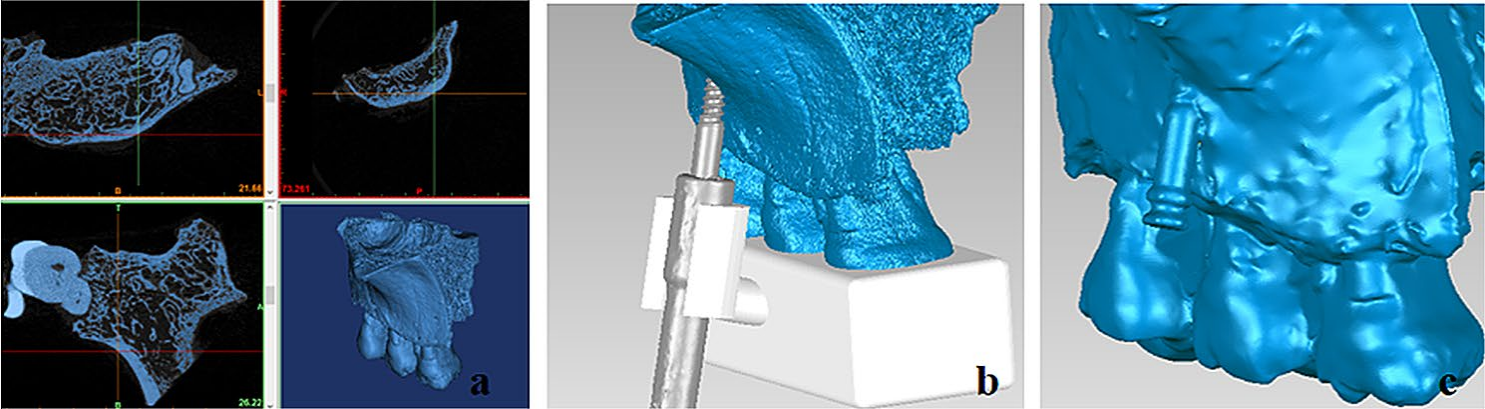
The digital model of screw-maxilla specimen. (**a**) Reconstruction of the maxilla specimen based on µCT images. (**b**) The insertion of mini-screws with the help of a guide plate (white model). (**c**) The scanning model of screw-maxilla specimen

Twelve fresh maxillae containing the infrazygomatic crest regions from six body donors were collected. Sectional images of the maxillae specimens were acquired using µCT-80 (Scanco Medical, Bassersdorf, Switzerland). The X-ray settings were 55 KVp, 145 µA, and 8 W. The voxel size was 60.0 μm, and the integration time was 200 ms. These images were reconstructed in Mimics 19.0 (Materialise, Leuven, Belgium) to obtain the bone 3D model (Fig. 5a). According to the preset insertion trajectories, two mini-screws were inserted at the optimal and poor angulations into the infrazygomatic crest regions with similar cortical bone thickness on both sides of the maxilla from the same donor using the guide plates (Fig. 5b). The optimal and poor angulations to the bone surface were defined according to the results of the synthetic-bone test. These screw-bone specimens were scanned again using SmartScan to obtain screw-bone 3D models (Fig. 5c). The 3D models of screw-bone, mini-screw, and bone were also fitted in Geomagic Studio 2014. The SCA, STA, and EL were measured using the same method described in the synthetic-bone test.

**Fig. 6:**
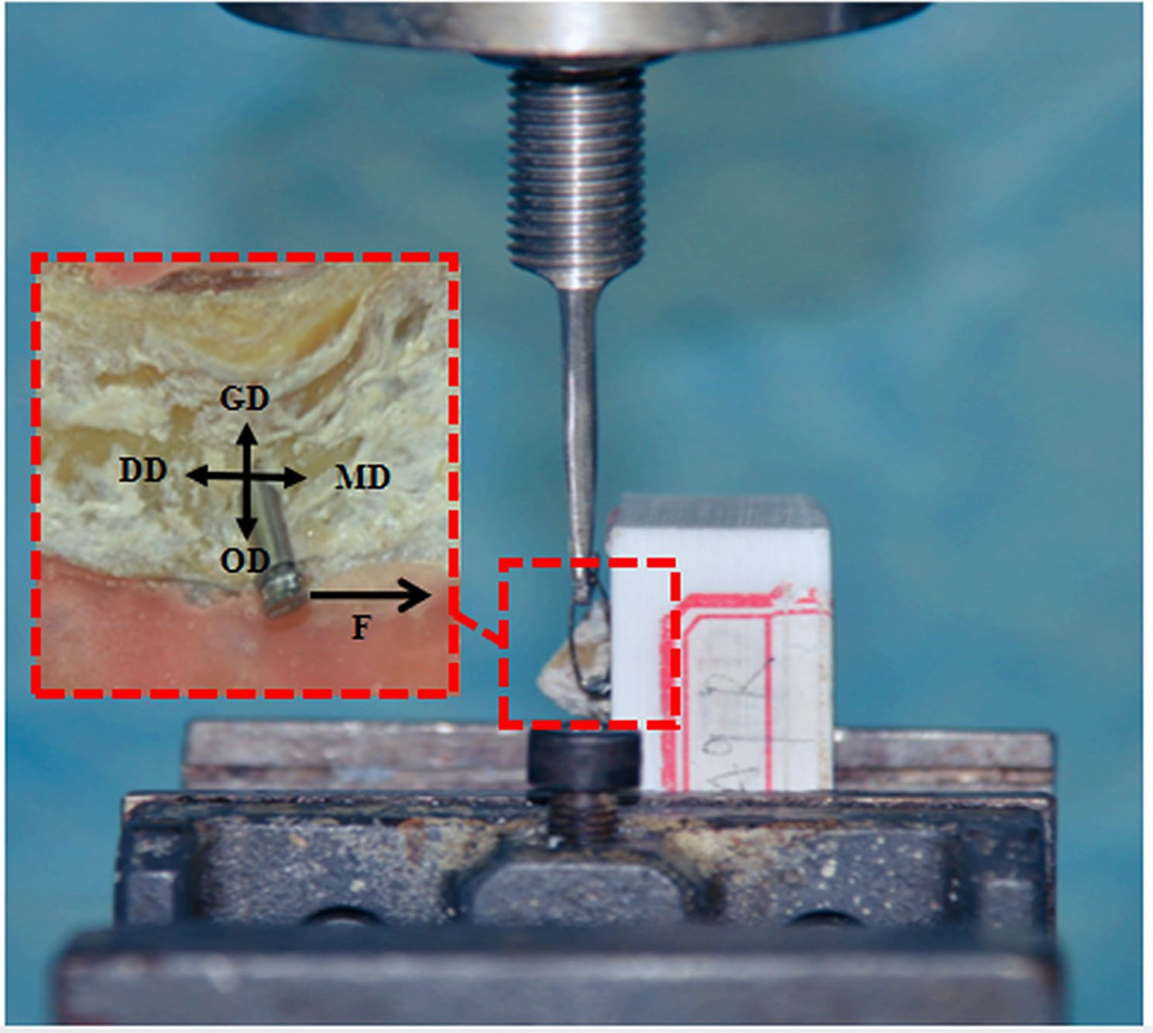
An image of lateral pull-out testing. *MD* mesial direction; *DD* distal direction; *GD* gingival direction; *OD* occlusal direction

The screw-bone specimens were cemented using polymethyl methacrylate and prepared for the subsequent lateral pull-out testing (Fig. 6). Lateral pull-out testing was conducted using the same method described in the synthetic-bone test.

### Statistical analysis

Statistical analysis was conducted using SPSS software (Version 20.0, IBM Corporation, Armonk, NY, USA) and SAS 9.4 for Windows (SAS Institute, Inc., Cary, USA). The Kruskal–Wallis H test was used for the synthetic-bone test to analyse differences in the LPS, SCA, STA, and EL among groups with the same insertion angles in the occlusogingival or mesiodistal directions, and a post hoc test with Bonferroni correction followed a significant finding. Spearman’s rank correlation analysis explored the correlations between the insertion angles and biomechanical metrics (LPS, SCA, STA, and EL). In addition, the correlations among these biomechanical metrics were explored using Spearman’s rank partial correlation analysis. A paired-sample *t*-test was used for the validation test to analyse the differences in LPS, SCA, STA, and EL between the poor and optimal angulations. Pearson’s partial correlation analysis explored the correlations among these biomechanical metrics.

Differences were considered statistically significant at *P*-values < 0.05 or Bonferroni-adjusted *P*-values  < 0.05. Bonferroni-adjusted *P*-value (*Adj*. *P*) = *P*-value _*_ n (n, number of comparisons).

## Results

### Results of the synthetic-bone test

For actual insertion angles in the occlusogingival and mesiodistal directions, the deviations between the actual and expected insertion angles were 1.97 (1.12, 3.17) and 2.00 (1.01, 3.05), respectively.

Significant differences were noted in the LPS, SCA, STA, and EL at different insertion angles in the occlusogingival direction among the groups with the same insertion angle in the mesiodistal direction (all *P* < 0.01). Significant differences were also observed in the SCA and STA at different insertion angles in the mesiodistal direction among the 40° and 50° groups in the occlusogingival direction (all *P* < 0.05). A significant difference was observed in the EL at different insertion angles in the mesiodistal direction only in the 40° occlusogingival direction (*P* = 0.001). The LPS, SCA, STA, and EL for each group and the results of multiple comparisons among groups at the same insertion angles in the occlusogingival or mesiodistal directions are listed in Tables 1, 2, 3 and 4, respectively.

**Table 1:** Comparison of the maximum LPS (N) of mini-screws for nine combinations of occlusogingival and mesiodistal angles

Insertion Angle		Mesiodistal direction					
		*90°*		*75°*		*60°*	
Occlusogingival direction	*30°*	50.24^d, g^ (45.66, 55.11)	51.13 ± 5.99	52.76^e, h^ (47.72, 56.99)	53.58 ± 10.64	51.16^f, i^ (46.06, 62.77)	53.83 ± 8.86
	*40°*	76.68^a^ (72.67, 83.43)	77.16 ± 6.43	81.37^b^ (69.70, 100.20)	83.86 ± 14.98	78.39^c^ (69.41, 107.63)	84.41 ± 18.42
	*50°*	109.87^a^ (93.33, 124.74)	109.94 ± 20.86	101.97^b^ (92.07, 121.20)	106.87 ± 18.98	120.85^c^ (90.87, 124.69)	113.28 ± 18.47

*NOTE*: Multiple comparisons of the LPS (N) have only been conducted among groups in the same row (in the same occlusogingival direction) or column (in the same mesiodistal direction)

Superscript letters indicated a statistically significant difference between the group that the cell represents and the group that the letter represents in the post hoc test with Bonferroni correction (Bonferroni-adjusted *P*-value < 0.05)

*LPS*: lateral pull-out strength

a: 30°/90° group; b: 30°/75° group; c: 30°/60° group

d: *40°/*90° group; e: *40°/75°* group; f: *40°/60°* group

g: *50°/90°* group; h: *50°/75°* group; i: *50°/60°* group

**Table 2:** Comparison of the SCA (mm^2^) of mini-screws for nine combinations of occlusogingival and mesiodistal angles

Insertion Angle		Mesiodistal direction					
		*90°*		*75°*		*60°*	
Occlusogingival direction	*30°*	16.34 ^d, g^ (15.94, 17.01)	16.44 ± 0.66	16.93 ^e, h^ (16.53, 17.27)	16.94 ± 0.50	16.83 ^f, i^ (16.58, 17.70)	17.09 ± 0.68
	*40°*	14.25 ^a, g, f^ (13.84, 14.61)	14.21 ± 0.44	14.02 ^b, h, f^ (13.98, 14.41)	14.16 ± 0.36	14.81 ^c, d, e, i^ (14.62, 15.23)	14.87 ± 0.32
	*50°*	12.06 ^a, d, i^ (12.01, 12.20)	12.08 ± 0.22	12.28 ^b, e, i^ (12.10, 12.59)	12.34 ± 0.26	13.03 ^c, g, h, f^ (12.80, 13.22)	13.03 ± 0.28

*NOTE*: Multiple comparisons of the SCA (mm^2^) have only been conducted among groups in the same row (in the same occlusogingival direction) or column (in the same mesiodistal direction)

Superscript letters indicated a statistically significant difference between the group that the cell represents and the group that the letter represents in the post hoc test with Bonferroni correction (Bonferroni-adjusted *P*-value < 0.05)

*SCA*: screw-cortical bone contact area

a: 30°/90° group; b: 30°/75° group; c: 30°/60° group

d: 40°/90° group; e: 40°/75° group; f: 40°/60° group

g: 50°/90° group; h: 50°/75° group; i: 50°/60° group

**Table 3:** Comparison of the STA (mm^2^) of mini-screws for nine combinations of occlusogingival and mesiodistal angles

Insertion Angle		Mesiodistal direction						
		*90°*		*75°*		*60°*		
Occlusogingival direction	*30°*	7.60 ^d, g^ (6.95, 8.54)	7.68 ± 1.27	7.09 ^e, h^ (6.35, 7.61)	7.06 ± 0.85	7.13 ^f, i^ (6.07, 8.59)	7.17 ± 1.48	
	*40°*	12.92 ^a, f^ (12.34, 13.82)	12.93 ± 1.04	11.54 ^b^ (10.68, 12.69)	11.57 ± 1.15	10.31^c, d, i^ (10.03, 10.84)	10.43 ± 0.56	
	*50°*	14.92 ^a, i^ (14.02, 15.80)	14.98 ± 1.05	14.11^b^ (13.29, 14.29)	13.87 ± 0.95	13.59 ^c, g, f^ (12.32, 14.19)	13.44 ± 1.04	

*NOTE*: Multiple comparisons of the STA (mm^2^) have only been conducted among groups in the same row (in the same occlusogingival direction) or column (in the same mesiodistal direction)

Superscript letters indicated a statistically significant difference between the group that the cell represents and the group that the letter represents in the post hoc test with Bonferroni correction (Bonferroni-adjusted *P*-value < 0.05)

*STA*: screw-trabecular bone contact area

a: 30°/90° group; b: 30°/75° group; c: 30°/60° group

d: *40°/*90° group; e: *40°/75°* group; f: *40°/60°* group

g: 50°/90° group; h: 50°/75° group; i: 50°/60° group

**Table 4:** Comparison of the EL (mm) of mini-screws for nine combinations of occlusogingival and mesiodistal angles

Insertion Angle		Mesiodistal direction					
		*90°*		*75°*		*60°*	
Occlusogingival direction	*30°*	8.41 ^d, g^ (8.35, 8.57)	8.43 ± 0.14	8.51 ^e, h^ (8.36, 8.62)	8.49 ± 0.15	8.41 ^i^ (8.17, 8.70)	8.43 ± 0.27
	*40°*	7.97 ^a, e, f^ (7.86, 8.09)	7.99 ± 0.13	8.10 ^b, d^ (8.04, 8.41)	8.20 ± 0.20	8.27 ^i, d^ (8.22, 8.32)	8.26 ± 0.07
	*50°*	7.96 ^a^ (7.84, 8.06)	7.94 ± 0.14	8.05 ^b^ (7.99, 8.16)	8.07 ± 0.15	8.02 ^c, f^ (7.94, 8.21)	8.05 ± 0.17

*NOTE*: Multiple comparisons of the EL (mm) have only been conducted among groups in the same row (in the same occlusogingival direction) or column (in the same mesiodistal direction)

Superscript letters indicated a statistically significant difference between the group that the cell represents and the group that the letter represents in the post hoc test with Bonferroni correction (Bonferroni-adjusted *P*-value < 0.05)

*EL*: exposure length

a: 30°/90° group; b: 30°/75° group; c: 30°/60° group

d: 40°/90° group; e: 40°/75° group; f: 40°/60° group

g: 50°/90° group; h: 50°/75° group; i: 50°/60° group

The LPS, SCA, STA, and EL had significant correlations with the angle in the occlusogingival direction (*r*_*LPS*_ = 0.886, *r*_*SCA*_ = -0.946, *r*_*STA*_ = 0.911, and *r*_*EL*_*=* -0.731, respectively; all *P* < 0.001). In contrast, the LPS, SCA, STA, and EL had no significant correlation with the angle in the mesiodistal direction (all *P* > 0.05).

When the SCA and EL were controlled for, a weak positive correlation was observed between the STA and LPS (*r*_*s*_ = 0.245, *P* = 0.022). However, when the STA and EL were controlled for, no statistically significant correlation was observed between the SCA and LPS (*r*_*s*_ = -0.069, *P* = 0.521). Moreover, when the STA and SCA were controlled for, no statistically significant correlation was observed between the EL and LPS (*r*_*s*_ = 0.110, *P* = 0.306).

### Results of the cadaver-validation test

For actual insertion angles in the occlusogingival and mesiodistal directions, the deviations between the actual and expected insertion angles were 5.44 ± 2.59 and 3.31 ± 1.92, respectively.

Based on the results of the synthetic-bone test, the occlusogingival angulation of 30° is the poor angle and 50° is the optimal angle under each angle in the mesiodistal direction. Therefore, at each angle in the mesiodistal direction (60°/75°/90°), two maxillae were respectively assigned for the occlusogingival angulation validation test.

**Fig. 7:**
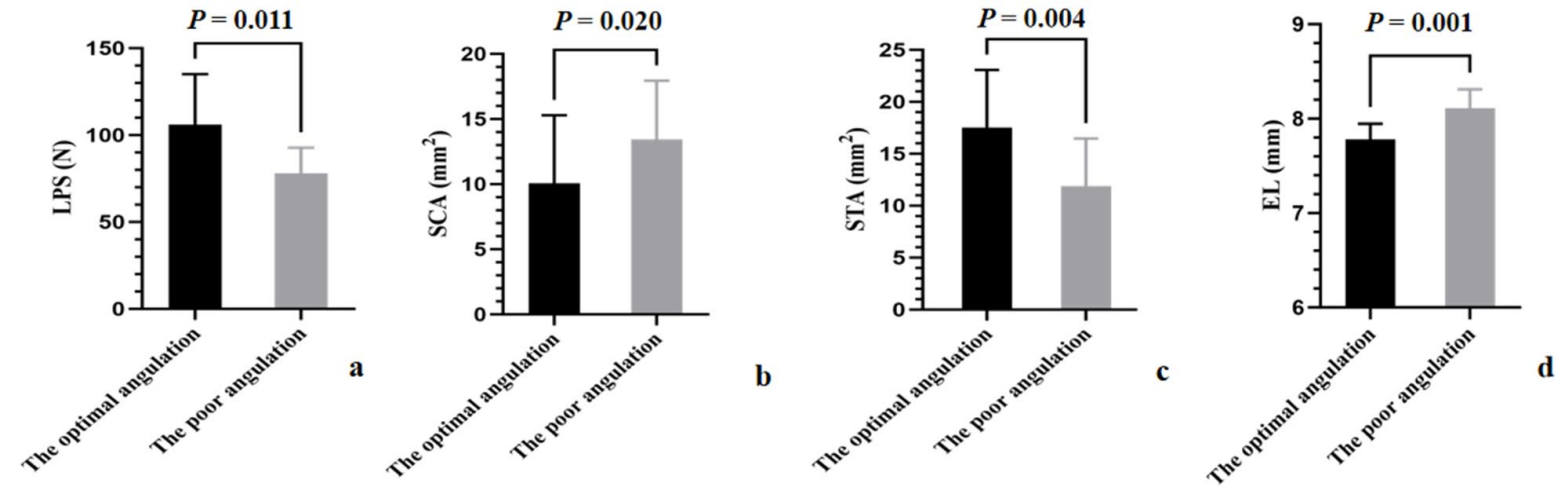
The differences between the optimal and poor angulations in cadaver-validation testing. (**a**) The difference in the LPS. (**b**) The difference in the SCA. (**c**) The difference in the STA. (**d**) The difference in the EL. *LPS* lateral pull-out strength; *SCA* screw-cortical bone contact area; *STA* screw-trabecular bone contact area; *EL* exposure length

The differences between the poor and optimal angulations are shown in Fig. 7. Statistically significant differences were observed between the two groups in LPS (*P* = 0.011), SCA (*P* = 0.020), STA (*P* = 0.004), and EL (*P* = 0.001).

When the SCA and EL were controlled for, a positive correlation was observed between the STA and LPS (*r* = 0.720, *P* = 0.019). However, when the STA and EL were controlled for, no statistically significant correlation was observed between the SCA and LPS (*r* = 0.566, *P* = 0.088). Moreover, when the STA and SCA were controlled for, no statistically significant correlation was observed between the EL and LPS (*r* = 0.570, *P* = 0.086).

## Discussion

The infrazygomatic crest mini-screw has been commonly used by orthodontists. Nevertheless, mobility of mini-screw at the initial stage can occur after insertion due to the insufficient primary stability, leading to the poor post-treatment satisfaction. Insertion angle is one of the key factors that could affect the primary stability, but there remains ambiguity regarding the biomechanical performance of the mini-screws at different insertion angles. In this study, we analysed the SCA, STA, EL, and LPS of infrazygomatic crest mini-screws inserted at different commonly used angles and explored their relationships. The results showed that the LPS was significantly correlated with the occlusogingival angulations and the STA significantly affected the LPS of the infrazygomatic crest mini-screw. Consequently, the null hypothesis was rejected.

In this study, we conducted tests on synthetic bone (polyurethane composite blocks) and natural bone (cadaver specimens). Synthetic bone has been widely used in in vitro studies because of its uniformity, consistent properties, and unrestricted and convenient sources [12, 17, 24]. Hence, we analysed the biomechanical metrics of the infrazygomatic crest mini-screw at all nine different insertion angles using synthetic bones to explore the trend in metric changes of the mini-screws at different angles. However, as the synthetic-bone test cannot precisely reproduce clinical insertion settings, further validation was needed on fresh cadaver specimens. In our study, the biomechanical metrics of the infrazygomatic crest mini-screw were analysed at the optimal and poor angulations in the cadaver-validation test.

Primary stability can be evaluated with quantitative methods, such as insertion torque (IT), resonance frequency analysis (RFA), and pull-out strength (PS). However, whether IT can predict screw retention in bone tissue is still controversial; thus, IT may not be an effective method for predicting mini-screw retention [25, 26]. As for RFA, the measured values in different studies were not comparable due to the lack of a standard transducer that matches the mini-screw [9, 27, 28]. Hence, the PS may be preferred to evaluate the primary stability. Because the orthodontic forces are applied in parallel to the surface of the cortical bone, and lateral loading might more closely mimic clinical orthodontic loading [29], the LPS was used to evaluate the primary stability of mini-screws in our study.

In clinical practice, the insertion direction of mini-screws is not one-dimensional. However, few studies have evaluated the influence of the insertion angles in both the occlusogingival and mesiodistal directions on the primary stability. Therefore, we measured the LPS of the mini-screws in these two directions. Because of the difficulties in insertion at the cross-combined angles, guide plates with different insertion angles were designed and 3D-printed to assist with mini-screw insertion. The deviation values between the actual and expected insertion angles were acceptable compared with those of the prior study [12].

In our study, we observed a significant positive correlation between occlusogingival angulation and LPS. This result is consistent with previous findings from Woodall et al. [30] who found that the maximum anchorage force of the mini-screw under tangential force also increased when the bone contact angle of the mini-screw increased from 30° to 60° and 90°. Moreover, we found no significant correlation between the insertion angle in the mesiodistal direction and LPS. The study from Lee et al. [31] supports these results; they found that there was no significant difference in stress distribution and displacement of the mini-screw between the insertion angles of 30°, 60°, and 90° in the mesiodistal directions when applying LPS (named L0° in their study). However, it should be noted that our study’s range of insertion angles only included the commonly used angles of the infrazygomatic crest mini-screw. Therefore, we cannot further explore the LPS changes when the insertion angle increased from 60° to 90°.

The cortical bone has been defined as a key factor affecting the primary stability of mini-screws at different insertion angles [20, 32]. Many researchers believed that the cortical bone thickness in contact with the mini-screw was negatively correlated with the occlusogingival angles [33] and positively correlated with the primary stability [12, 19]. The SCA, as the cortical bone area in contact with the mini-screw, should have shown the same correlation. However, only the same negative correlation between the SCA and angulations in the occlusogingival direction was shown in our study, and the positive correlation between the LPS and SCA was not observed. Extensive cortical bone micro-damage caused by oblique angulation (30°–50°) in the occlusogingival direction might be the reason for this difference [29, 34, 35].

The EL of the mini-screw also plays an important role in the primary stability, which could significantly impact bone stress around the mini-screw [18]. A negative correlation between EL and angulation in the occlusogingival direction was observed in this study. It seems that EL may affect the LPS of the mini-screws with different angles. However, further results indicated no statistically significant correlation between EL and the LPS of the mini-screws at different angles. The main reason for this difference may be the small ranges of EL variations under different insertion angles. In Lin et al.’s study, the distances of EL variations were 2 mm [18]. In contrast, the distances of EL variations at commonly used different angles were less than 1 mm in our study, which may not be sufficient to cause significant changes in the LPS.

The role of trabecular bone in the primary stability of mini-screws is usually ignored. Our study showed that the STA increased with increasing insertion angles in the occlusogingival direction. Moreover, we observed a positive correlation between the STA and LPS, which is consistent with the results reported by Marquezan et al. [15]. The insertion angle has an important effect on the trabecular bone stress, and the small effect on the cortical bone stress may be responsible for this result [18].

These findings were further verified in fresh cadavers in this study. Although the values of the biomechanical metrics differed from those in the cadaver-validation test, the comparisons between the poor and optimal angulations to the bone surface showed the same trend as the results of the synthetic bone test. Moreover, correlation analysis between the STA and LPS verified the importance of the STA in the primary stability of infrazygomatic crest mini-screws at different insertion angles. Therefore, the STA may be the preferred concern for the primary stability of the infrazygomatic crest mini-screw with a special insertion angle.

Considering these findings, it can be concluded that the greater the diameter and length of mini-screw, the greater the STA, and thus the better the mini-screw stability. However, screw diameter and length are commonly limited by the bone thickness and depth in the infrazygomatic crest region, due to the variability of anatomical structures [1, 36]. According to our previous study [22], the available bone thickness was negatively correlated with bone depth in the infrazygomatic crest region. Therefore, we suggest that orthodontists select a mini-screw with smaller diameter but longer length in the infrazygomatic crest region to achieve the better stability without structural damage. However, further research is still needed to determine the optimal size of infrazygomatic crest mini-screw.

It also should be noted that the results of this study need to be interpreted within the study’s limitations. These limitations included the utilization of in vitro models and limited insertion angles we explored. In the future, we intend to further analyse the effects of other factors (size of mini-screw, type of thread, direction of the applied force and so on) on the primary stability and conduct in vivo researches to evaluate the secondary stability of the mini-screw under different factors.

## Conclusions

The primary stability of the infrazygomatic crest mini-screw was correlated with occlusogingival angulations. The STA significantly affected the primary stability of the infrazygomatic crest mini-screw, but the SCA and EL did not.

## Data Availability

The datasets used and/or analyzed during the current study are available from the corresponding author on reasonable request.

## Abbreviations

EL: Exposure length
SCA: Screw-cortical bone contact area
STA: Screw-trabecular bone contact area
LPS: Lateral pull-out strength
